# Simultaneous Development and Validation of an HPLC Method for the Determination of Furosemide and Its Degraded Compound in Pediatric Extemporaneous Furosemide Oral Solution

**DOI:** 10.3390/molecules30194031

**Published:** 2025-10-09

**Authors:** Katsanee Srejomthong, Thanawat Pattananandecha, Sutasinee Apichai, Suporn Charumanee, Busaban Sirithunyalug, Fumihiko Ogata, Naohito Kawasaki, Chalermpong Saenjum

**Affiliations:** 1 Department of Pharmaceutical Sciences, Faculty of Pharmacy, Chiang Mai University, Chiang Mai 50200, Thailand; katsanee.s@cmu.ac.th (K.S.); chsuporn@gmail.com (S.C.); busaban.s@cmu.ac.th (B.S.); 2 Research Center for Innovation in Analytical Science and Technology for Biodiversity-Based Economic and Society (I-ANALY-S-T_B.BES-CMU), Multidisciplinary Research Institute (MDRI), Chiang Mai University, Chiang Mai 50200, Thailand; thanawat.pdecha@gmail.com (T.P.); sutasinee.apichai@gmail.com (S.A.); 3 Office of Research Administration, Chiang Mai University, Chiang Mai 50200, Thailand; 4 Faculty of Pharmacy, Kindai University, 3-4-1 Kowakae, Higashi-Osaka 577-8502, Osaka, Japan; ogata@phar.kindai.ac.jp (F.O.); kawasaki@phar.kindai.ac.jp (N.K.); 5 Antiaging Center, Kindai University, 3-4-1 Kowakae, Higashi-Osaka 577-8502, Osaka, Japan

**Keywords:** furosemide, furosemide related compound B, methyl paraben, propyl paraben, HPLC, extemporaneous formulation

## Abstract

Furosemide (FUR) is a loop diuretic widely used in pediatric care. However, no standardized oral liquid formulation exists due to degradation concerns, particularly the formation of furosemide-related compound B (FUR-B). This study aimed to develop and validate the HPLC method for the simultaneous quantification of FUR, FUR-B, methylparaben (MP), and propylparaben (PP) in pediatric extemporaneous oral solutions. Chromatographic separation was achieved using a Symmetry^®^ C18 column (4.6 × 250 mm, 5 µm) with a mobile phase of 0.1% acetic acid in water and acetonitrile (60:40, *v*/*v*) at 1.0 mL/min of flow with injection volume at 10 µL. Detection at 272 nm provided optimal sensitivity, especially for low concentrations of FUR-B. Forced degradation confirmed baseline separation of FUR from its degradation products. The condition showed high linearity (R^2^ > 0.995), accuracy (recoveries 98.2–101.0%), and precision (RSD ≤ 2%). Robustness and ruggedness tests under varied conditions, analysts, and intra-day yielded consistent performance. Application to extemporaneous formulations showed that refrigeration (2–8 °C) retained initial composition, while elevated temperatures (30 °C and 40 °C) promoted FUR degradation, with FUR-B increasing to 6.84% after 90 days and greater MP and PP degradation. This validated method offers a reliable analytical tool for monitoring chemical changes and supporting quality control of pediatric FUR extemporaneous formulations.

## 1. Introduction

Congestive heart failure (CHF) is a complex clinical syndrome resulting from structural or functional abnormalities of the heart, impairing its ability to fill with and pump blood effectively. Diuretics are the main class of drugs used to reduce fluid overload in the circulatory system and lungs, with loop diuretics being the most commonly prescribed for CHF management. [1]. Furosemide (FUR) is widely administered intravenously at doses of 1–2 mg/kg or 1–2 mg/h. For long-term use, a dosage of 1–4 mg/kg is recommended [2]. FUR is an effective and safe medication for the treatment of hypertension and vascular diseases. However, its pharmacokinetics and pharmacodynamics can vary significantly across different age groups within the pediatric population. This variability arises due to physiological differences in organ and skin development, metabolism, and other factors, ranging from preterm neonates to adolescents [3,4]. Given these physiological differences, the formulation of FUR for pediatric use should carefully balance risks and benefits. Key considerations in pediatric formulation design involve developing age- and weight-adjustable dosage forms that are well-accepted, palatable, easy to administer, and, most importantly, both safe and effective. The goal is to create a single formulation that can be safely used across a broad age range, ensuring optimal therapeutic outcomes while accommodating the diverse needs of pediatric patients [5,6].

However, there is currently no suitable oral liquid formulation of FUR for pediatric patients and individuals with swallowing difficulties. According to the United States Pharmacopeia (USP), furosemide oral solution must contain not less than 90.0% and not more than 110.0% of the labeled active ingredient, with a pH range of 7–10 and a maximum allowable limit of 1.5% for furosemide-related compound B (FUR-B) [7]. Extemporaneous preparation of furosemide oral solution requires quantitative analysis to ensure quality control and safety. FUR is highly light-sensitive, and its related compound, FUR-B, is a degradation product and impurity of the drug [8,9]. The presence of FUR-B is concerning, as it may contribute to reduced drug efficacy and potential toxicity [10]. Additionally, extemporaneous formulations often include preservatives such as methylparaben (MP) and propylparaben (PP) to enhance stability and prevent microbial contamination [11]. According to EMA (2015), the acceptable daily intake (ADI) for methylparaben (MP) and propylparaben (PP) in children is 10 and 2 mg/kg/day, respectively. At typical concentrations used in oral formulations (0.2% for MP and 0.06% for PP), estimated intakes remain within or close to these limits [12]. In practice, MP and PP are combined in oral formulations at 0.015–0.2% and 0.02–0.06%, respectively, to balance safety with antimicrobial efficacy. However, few studies have assessed the stability and shelf-life of extemporaneous furosemide oral solutions containing these preservatives. A review of the literature developed an HPLC method to analyze FUR and MP in furosemide oral solution for stability assessment. The method used an isocratic system with detection at 270 nm but could not quantify FUR-B [13]. Giannetti et al. [9] optimized an HPLC method for furosemide tablet degradation using 0.1% formic acid in water and acetronitrile (ACN) in the ratio of 70:30 as a mobile phase, detecting FUR-B and FUR at 3.25 and 11.24 min at 272 nm, respectively. However, the study did not include method validation, and the analysis of FUR-B was only semi-quantitative. Xu et al. [14] investigated FUR impurities using a gradient-phase HPLC method, achieving separation but requiring longer analysis times. Thean et al. [15] studied furosemide suspension stability using different HPLC conditions to quantify FUR and its impurity, detecting FUR-B at 254 nm. Based on the reviewed literature, there is currently no established HPLC method for the simultaneous quantification of FUR, FUR-B, MP, and PP.

This study aims to develop and validate an HPLC method for the simultaneous determination of four compounds, including FUR, FUR-B, MP, and PP, employing a more widely used column type in place of the less commonly utilized USP-recommended option [7] while maintaining high resolution, sensitivity, and specificity. The method also allows for accurate quantification of MP and PP at levels relevant to pediatric safety and achieves shorter retention times compared to previously reported methods, offering a practical and reliable approach for pharmaceutical quality control laboratories.

## 2. Results

### 2.1. System Suitability

**Figure 1:**
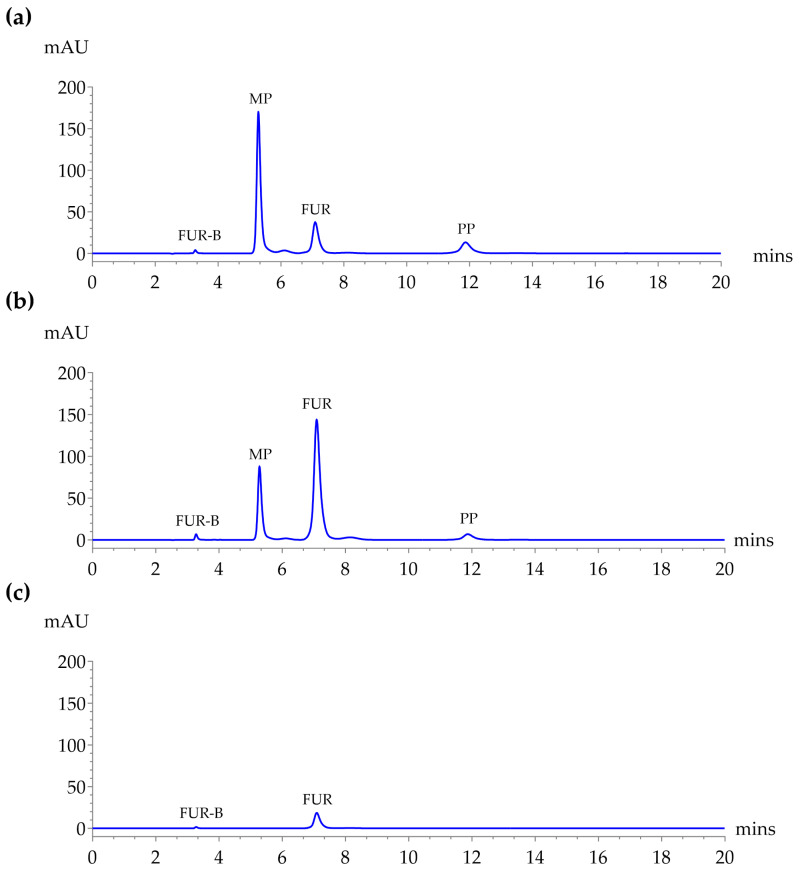
HPLC chromatograms of a standard mixture containing FUR (50 µg/mL), FUR-B (1.0 µg/mL), MP (10 µg/mL), and PP (2.0 µg/mL) using a Symmetry^®^ C18 column at 25 °C using a mobile phase of 0.1% acetic acid in DI water:ACN (60:40, *v*/*v*) at a flow rate of 1.0 mL/min with an injection volume of 10 µL. Detection at (**a**) 254 nm, (**b**) 272 nm, and (**c**) 330 nm.

A comparison of detection wavelengths at 254, 272, and 330 nm was conducted to determine the optimal wavelength for simultaneous analysis of FUR, FUR-B, MP, and PP. As shown in Figure 1, although 254 nm provided slightly higher peak areas for MP and PP, 272 nm offered superior sensitivity for FUR-B and FUR, which are critical analytes for stability assessment in extemporaneous formulation, especially considering FUR-B’s low concentration. At 330 nm, MP and PP were not detectable. Therefore, 272 nm was selected as the most appropriate wavelength for subsequent analysis, balancing sensitivity, and comprehensive detection. Given the importance of detecting FUR-B, a degradation product typically present at low concentrations, 272 nm was prioritized despite the slightly lower response for MP and PP.

**Figure 2:**
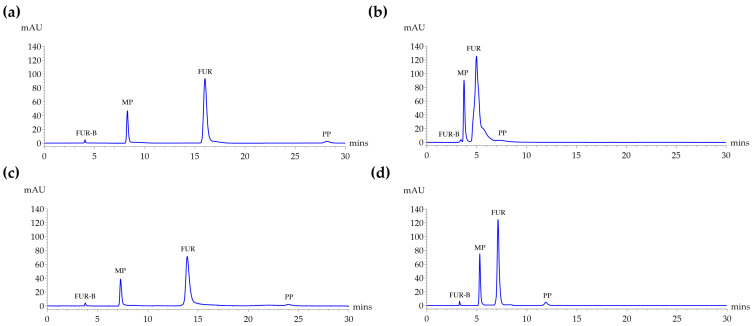
HPLC chromatograms of standard mixtures containing FUR (50 µg/mL), FUR-B (1.0 µg/mL), MP (10 µg/mL), and PP (2.0 µg/mL) detected at 272 nm under four analytical conditions: (**a**) Kinetex C18 with DI water:ACN (70:30, *v*/*v*) at a flow rate of 0.5 mL/min, (**b**) Kinetex C18 with DI water:ACN (60:40, *v*/*v*) at a flow rate of 0.5 mL/min, (**c**) Symmetry^®^ C18 with DI water:ACN (70:30, *v*/*v*) at a flow rate of 1.0 mL/min, and (**d**) Symmetry^®^ C18 with DI water: acetonitrile (60:40, *v*/*v*) at a flow rate of 1.0 mL/min. All analyses were performed at a column temperature of 25 °C with an injection volume of 10 µL.

Four conditions combining two column types, namely Kinetex C18 (Phenomenex, Torrance, CA, USA) and Symmetry^®^ C18 (Waters Corporation, Milford, MA, USA), and two mobile phase compositions, including 0.1% glacial acetic acid in DI water:ACN in the ratio of 70:30 and 60:40 *v*/*v*, were tested using standard mixtures of FUR-B, MP, FUR, and PP at a detection wavelength of 272 nm as shown in Figure 2.

As shown in Table 1, the highest resolution was obtained using the Kinetex C18 column with a mobile phase consisting of 0.1% acetic acid in DI water:ACN in the ratio of 70:30 at a flow rate of 0.5 mL/min. Under this condition, high resolution values were observed between analyte peaks, MP (31.68 min), FUR (30.12 min), and PP (45.44 min), demonstrating effective separation. However, using a 0.1% acetic acid in DI water:ACN in the ratio of 60:40 as mobile phase under the same column and flow rate resulted in a substantial decline in resolution and an increase in tailing. In particular, the resolution between FUR-B and MP (Rs = 1.85) was insufficient, failing to meet the acceptance criteria. When using the Symmetry^®^ C18 column at 1 mL/min, it also showed acceptable resolution, though lower than that of Kinetex C18. Under 0.1% acetic acid in DI water:ACN in the ratio of 70:30, the resolution between MP/FUR (20.77 min) and FUR/PP (21.43 min) was better, but peak tailing was more prominent, while with 0.1% acetic acid in DI water:ACN in the ratio of 60:40, resolution values further declined and the symmetry factors (As) slightly improved compared to the 70:30 condition.

Retention times of all analytes varied with mobile phase composition and column type, which are shown in Table S1. Increasing the proportion of ACN in the mobile phase from 30% to 40% led to significantly shorter retention times for all targeted analytes. The Symmetry^®^ C18 column with a mobile phase of 0.1% acetic acid in DI water:ACN (60:40, *v*/*v*) offered a suitable and practical method for the simultaneous analysis of the four targeted compounds. Under this condition, resolution values for all analytes fell within the acceptance value of >2.0, as recommended by the Center for Drug Evaluation and Research at the Food and Drug Administration [16]. Moreover, the Symmetry^®^ C18 (60:40) method provided significantly shorter retention times, making it more suitable for routine quality control applications where analytical efficiency is essential. Therefore, Symmetry^®^ C18 (60:40) was chosen for further analysis, balancing chromatographic performance and analytical throughput.

### 2.2. The Forced Degradation Studies

Chromatograms demonstrated baseline separation of FUR from its degradation products, as illustrated in Figure S1. The extent of FUR degradation, the formation of degradation products, mass balance, and peak purity index are summarized in Table 2. Photodiode array (PDA) peak-purity assessment verified that the FUR peak remained spectrally pure and chromatographically resolved from all related degradation species across all stress conditions. Mass balance values (calculated as the sum of assay and degradation products relative to the initial concentration) were within acceptable limits, confirming that the developed method is stability-indicating.

### 2.3. Method Validation

The correlation coefficients (R^2^) for FUR, FUR-B, MP, and PP were all greater than 0.995, indicating excellent linearity across the tested concentration range. In addition, the assessment of analytical sensitivity showed that all analytes exhibited acceptable limits of detection (LOD) and limits of quantification (LOQ) following established pharmaceutical standards, as summarized in Table 3.

The accuracy of the analytical method refers to the closeness of the measured values to the true values. In this study, recovery values for all analytes ranged from 98.8% to 101.0% for intra-day analysis and from 98.2% to 100.9% for inter-day analysis. These values fall within the generally accepted range of 98–102%, indicating that the method is accurate [17]. Precision was assessed by the RSD. According to ICH guidelines [18], RSD should not exceed 2%. The results demonstrated RSDs of 0.1–1.5% (repeatability) and 0.50–2.0% (intermediate precision), with most values well below 2%, confirming the method’s precision. Overall, the analytical method showed acceptable accuracy and precision across all tested concentrations, as summarized in Table 4.

The robustness and ruggedness of the selected method (Symmetry^®^ C18, 60:40 mobile phase) was evaluated by deliberately varying key chromatographic parameters. For robustness, small variations in column temperature (23 and 27 °C compared to nominal 25 °C), flow rate (0.9 and 1.1 mL/min compared to nominal 1.0 mL/min), and detection wavelength (270 and 274 nm compared to nominal 272 nm) showed minimal impact on method performance. For ruggedness, analyses were performed by different analysts and on different instruments, with all results showing consistent performance. RSD values for peak areas remained below 2% for all analytes under varied conditions, with symmetrical factors consistently within acceptable ranges (0.8–1.8). Theoretical plate counts remained stable (>9000 for all compounds), confirming the method’s reliability for routine use (Tables S3 and S4).

### 2.4. Application of the Validated HPLC Method

**Figure 3:**
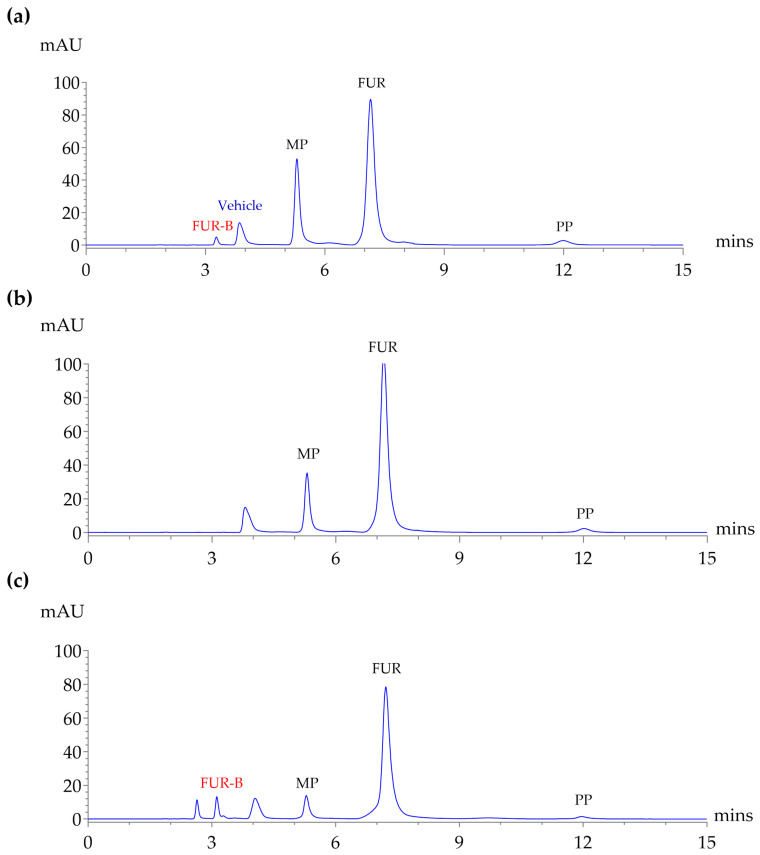
HPLC chromatograms show (**a**) standard mixture of FUR, FUR-B, MP, and PP in vehicle solution; (**b**) extemporaneous FUR oral solution at the initial time point (day 0); and (**c**) extemporaneous FUR oral solution after 90 days of storage at 40 °C. Analyses were performed using the validated HPLC method.

As shown in Figure 3, the validated HPLC method was applied to analyze an extemporaneously prepared furosemide oral solution containing FUR, MP, and PP as formulation components, which had been stored at 2–8 °C, 30 ± 2 °C, and 40 ± 2 °C/75% RH for 0, 7, 14, 21, 30, 45, 60, 75, and 90 days. The objective was to demonstrate the suitability and reliability of the validated method for routine analysis of extemporaneous preparations. FUR-B, as an impurity, was quantified to show the method’s ability to detect minor components. The total run time was less than 15 min, demonstrating good resolution, sharp peak shapes, and minimal tailing. The results obtained from the method showed satisfactory consistency and reliability.

**Figure 4:**
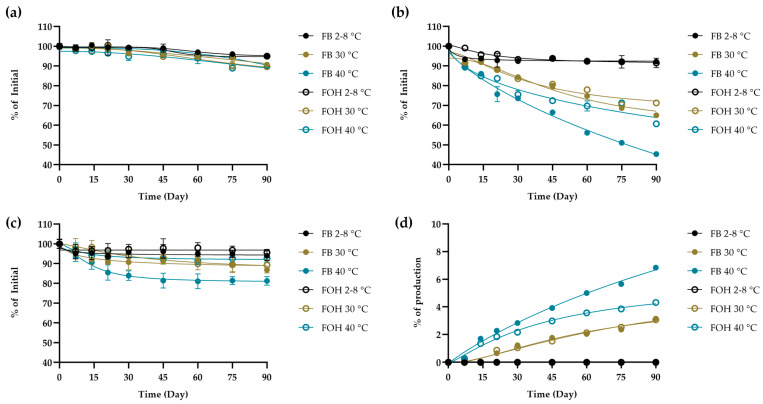
Chemical assessment of (**a**) FUR, (**b**) MP, and (**c**) PP, as well as the formation of the degradation product (**d**) FUR-B, in phosphate buffer (FB) and hydroxide-containing (FOH) formulations under storage conditions of 2–8, 30, and 40 °C.

As shown in Figure 4 (sigmoidal curve fit with 95% confidence intervals), the validated HPLC method was applied to extemporaneously prepared furosemide oral solutions containing FUR, MP, and PP stored under refrigerated conditions (2–8 °C) for up to 90 days to demonstrate its applicability. Under refrigerated conditions (2–8 °C) for both FB and FOH formulations, the chromatographic profiles showed only minor changes over time, whereas at higher storage temperatures (30 and 40 °C) the method detected a gradual decrease in the main components together with a progressive appearance of the FUR-B and a slight reduction in preservatives. These qualitative trends demonstrate the method’s capability to detect potential compositional changes without constituting a formal stability evaluation. Moreover, the data showed consistently well-resolved peaks for FUR, FUR-B, MP and PP. The assay produced reproducible quantitation and acceptable system-suitability values across all sampling points (Table S5), confirming its reliability for routine analysis.

Microbial assessment of the extemporaneous furosemide oral solution was performed at 0, 30, 60, and 90 days of storage using validated in-house protocols. The results demonstrated that the total aerobic microbial count (TAMC) did not exceed the acceptance limit of 200 CFU/mL, the total yeast and mold count remained below the criterion of 20 CFU/mL, and no contamination with the tested pathogens (*Staphylococcus aureus*, *Pseudomonas aeruginosa*, *Bacillus subtilis*, *Candida albicans*, *Aspergillus brasiliensis*, *and Escherichia coli*) was detected.

The pH of each formulation was monitored over 90 days under different storage conditions. Initial values were 8.03 ± 0.01 (FB) and 8.04 ± 0.03 (FOH), with a gradual decrease observed, remaining within the acceptance range of 7.0–10.0 (Table S6). After 90 days of refrigerated storage, pH values were 7.94 ± 0.01 (FB) and 7.83 ± 0.01 (FOH). The FB formulation exhibited minimal pH drift (−0.09 units at 2–8 °C to −0.21 units at 40 °C), whereas FOH showed a more pronounced decline (−0.21 units at 2–8 °C to −0.58 units at 40 °C).

## 3. Discussion

FUR is a key diuretic used to manage congestive heart failure, especially in pediatric patients, where dosing and formulation must consider developmental differences. Despite its widespread use, there is no standardized oral liquid formulation suitable for children, partly due to challenges in ensuring stability, efficacy, and safety, including control of degradation product FUR-B and preservative levels. This study developed a validated HPLC method capable of simultaneously quantifying FUR, FUR-B, and common preservatives, addressing a critical gap in quality control for pediatric formulations.

The USP recommends an L10 column and detection at 200–400 nm for FUR oral solution analysis [7]. In this study, a more widely used L1 (C18) column was employed due to its broad applicability and established use in furosemide analysis [9,19,20,21]. FUR exhibits multiple UV absorption maxima depending on the solvent used, with other studies reporting use of 230–283 nm, depending on the analytes of interest [22]. Under alkaline conditions (0.1 N NaOH), FUR demonstrates dual absorption maxima at 226 nm and 272 nm [23], FUR-B shows maximum absorption at 272 nm [9], indicating similar chromophoric behavior to the parent compound. The preservative compounds MP and PP exhibit characteristic UV absorption maxima at 258 nm [24] and 257 nm [25], respectively, in alcoholic solutions. According to USP, MP and PP are specified at 272 nm [26,27].

In this study, detection at 272 nm provided better sensitivity for FUR and FUR-B compared to 254 nm, which favored MP and PP. At 330 nm, preservatives were undetectable. Thus, 272 nm was selected as optimal, prioritizing the detection of FUR-B. Notably, several studies have also adopted 272 nm for FUR analysis [9,19]. While the sensitivity of MP and PP at 272 nm is indeed lower than at 254 nm, the method demonstrates sufficient analytical performance for preservative monitoring in real-world applications. At a 100-fold sample dilution, the LOQs correspond to 96% and 88% degradation of MP and PP, respectively, which remain well below the actual degradation observed at 90 days (55% for MP and 19% for PP). Thus, preservative quantification over the 90-day storage period is accurate. For extended long-term assessments beyond 90 days, reliable quantification can still be maintained under the same dilution, and additional sensitivity could be achieved, if necessary, by reducing the dilution factor. Therefore, the use of 272 nm does not adversely impact the accuracy of MP and PP determination, even at low concentrations relevant to microbiological safety.

**Figure 5:**
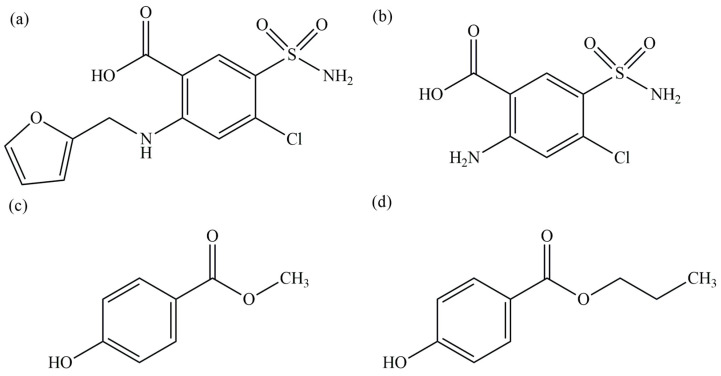
Chemical structures of the analyzed compounds: (**a**) Furosemide (FUR), (**b**) Furosemide-related compound B (FUR-B), (**c**) Methylparaben (MP), and (**d**) Propylparaben (PP).

Among the tested conditions, the Kinetex C18 column combined with 0.1% acetic acid in DI water:ACN (70:30) at 0.5 mL/min provided the best chromatographic performance, yielding high resolution (Rs > 30) and nearly symmetrical peaks (As ~1). This may be attributed to its core–shell particle technology, which enhances separation efficiency and peak sharpness by minimizing band broadening. However, FUR-B consistently exhibited peak fronting (As < 1.0) under both test conditions when analyzed on the Kinetex C18 column, with As of 0.73 and 0.80, in contrast to the Symmetry^®^ C18 column. Notably, use of the Kinetex C18 column with 0.1% acetic acid in DI water:ACN (70:30, *v*/*v*) resulted in an asymmetry factor (As) of 0.73, which is below the acceptable range of 0.8–1.8 [28]. This may be attributed to the Kinetex C18’s higher sensitivity to polar compounds, as FUR-B is the most polar among the compounds analyzed, as seen in its structure in Figure 5. However, the Symmetry^®^ C18 column, which uses fully porous particles, showed acceptable resolution under the same mobile phase but with more prominent peak tailing. Increasing the ACN content to 40% improved run time (all analytes eluted within 15 min) and slightly improved symmetry, though with some reduction in resolution. Nevertheless, all parameters remained within pharmacopeial limits (As 0.8–1.8 and Rs > 2.0 [16,28,29]). Considering its balance between acceptable resolution, shorter run time, and suitability for routine analysis, the Symmetry^®^ C18 column with DI water: ACN (60:40) was selected for the final method.

Forced degradation studies demonstrated that the validated HPLC method could effectively separate FUR from its degradation products under various stress conditions. Photodiode array peak-purity assessment confirmed that the FUR peak remained spectrally pure and fully resolved from all related species, indicating high specificity. Mass balance calculations, based on the sum of assay and degradation products relative to the initial concentration, were within acceptable limits, supporting the quantitative reliability of the method. These results confirm that the method is suitable for monitoring chemical changes and accurately quantifying FUR and its degradation products in extemporaneous oral formulations.

This study firstly presented the development and validation of an HPLC method for the simultaneous determination of four compounds (FUR, FUR-B, MP, and PP), employing a widely available column type instead of the less commonly used USP-recommended option. Compared with previously reported HPLC methods for FUR [9,13,14], the developed method advances from semi-quantitative to fully quantitative analysis, offering shorter retention times while maintaining high resolution, sensitivity, and specificity. The developed method demonstrated high linearity for all analytes, with R^2^ exceeding 0.995 across the tested concentration ranges. Sensitivity was also acceptable, with LOD and LOQ values meeting pharmaceutical standards. In terms of accuracy, recovery values for both intra- and inter-day analyses ranged from 98.2% to 101.0%, falling well within the generally accepted range (98–102%). Precision was confirmed by low RSD values, mostly below 2%, in line with ICH guidelines [17,18]. The result demonstrated the accuracy and precision of the validated method for simultaneous quantification of FUR, FUR-B, and preservatives. It provides a reliable, practical tool for pharmaceutical laboratories and addresses a critical gap in quality control of extemporaneous preparations. The robustness and ruggedness of the method were further evaluated by introducing small variations in chromatographic parameters, such as column temperature, flow rate, and detection wavelength, as well as by performing intra-day analyses with different analysts and instruments. The method showed minimal impact from these variations, with consistent peak shapes and reproducible results. These findings confirm that the validated HPLC method is not only accurate and precise under nominal conditions but also reliable and practical under typical laboratory variations, supporting its suitability for routine quality control of extemporaneous FUR oral solutions.

Furosemide is known to be chemically unstable, particularly in aqueous solutions where it degrades via hydrolysis into saluamine (CSA) and furfuryl alcohol. It also undergoes photodegradation through dechlorination, decarboxylation, and photo-hydrolysis, even under anaerobic conditions. Factors such as low pH, light exposure, and elevated temperature accelerate its degradation [9,30]. Due to concerns about degradation, the United States Pharmacopeia (USP) specifies that the amount of its main degradation product, FUR-B (CSA), must not exceed 1.5% of the labeled amount in finished products [7]. The validated HPLC method was successfully applied to monitor the chemical composition of an extemporaneous pediatric FUR oral solution stored under various conditions. The method provided sharp, well-resolved peaks with a run time under 15 min, enabling efficient and reliable quantification of all analytes, including the degradation product FUR-B. Results confirmed that FUR, MP, and PP remained stable under refrigerated conditions (2–8 °C), with no detectable FUR-B. However, storage at elevated temperatures (30 °C and 40 °C, 75% RH) led to time-dependent FUR degradation and FUR-B formation, especially at 40 °C, where FUR-B reached 6.84% by day 90. MP showed greater thermal degradation than PP, declining to ~45% at 40 °C, potentially affecting preservative efficacy [31]. Although preservative levels decreased over time, preliminary microbiological assessments under the conditions of this study showed that total aerobic microbial counts and yeast/mold levels stayed at acceptable levels throughout the 90-day storage period, and no *E. coli* was detected. These findings suggest that the residual MP and PP may have contributed to maintaining acceptable microbiological quality under the tested conditions. Current recommendations advise that extemporaneously prepared oral liquids be stored at 2–8 °C and used within 14 days if no preservative is present or within 35 days if a preservative is included, in the absence of established data. Within the specific conditions of the present work, the formulation maintained acceptable chemical and microbiological quality for up to 90 days under refrigeration; further confirmatory studies following regulatory guidelines would be needed before any extension of the assigned beyond-use date. pH monitoring revealed a temperature-dependent decrease within each formulation. However, at comparable storage conditions, the FB formulation exhibited less pH drift than FOH, reflecting a greater buffering capacity. Overall, the pH of both formulations remained within the USP acceptance range (7.0–10.0) [7] throughout the 90-day study at all storage temperatures (25, 30, and 40 °C).

## 4. Materials and Methods

### 4.1. Chemicals and Reagents

Furosemide (CAS No. 54-31-9) was obtained from Merck (St. Louis, MO, USA), while Furosemide related compound B (2-Amino-4-chloro-5-sulfamoylbenzoic acid) (CAS No. 3086-91-7) was purchased from Ambeed, Inc. (Arlington Heights, IL, USA. MP (CAS No. 99-76-3) and PP standards (CAS No. 94-13-3) were provided by the Reference Standard Center, Bureau of Drug and Narcotic (Nonthaburi, Thailand). Furosemide raw material (CAS 54-31-9) was sourced from IPCA Laboratories Limited (Mumbai, Maharashtra, India). MP (CAS No. 99-76-3) and PP (CAS No. 94-13-3) were also obtained from B.L.HUA (Bangkok, Thailand). Other excipients, including glycerin (CAS No. 56-81-5), propylene glycol (CAS No. 57-55-6), and saccharin sodium (CAS No. 128-44-9), were supplied by S. Tong Chemicals Co., Ltd. (Bangkok, Thailand). Glacial acetic acid (CAS No. 64-19-7) was purchased from Merck (Darmstadt, Germany). Acetonitrile (CAS No. 75-05-8) and sodium hydroxide (CAS No. 1310-73-2) were obtained from RCI Labscan Limited (Bangkok, Thailand). Sodium dihydrogen phosphate dihydrate (CAS No. 13472-35-0) was sourced from PanReac AppliChem (Barcelona, Spain), while disodium hydrogen phosphate (CAS No. 7558-79-4) was obtained from Qrec (Auckland, New Zealand).

### 4.2. HPLC System and Method Optimization

The analysis was performed using an HP 1200 Series liquid chromatography system (Agilent Technologies, Santa Clara, CA, USA). Two different columns—Kinetex C18 (2.6 µm, 4.6 × 150 mm) and Symmetry^®^ C18 (5 µm, 4.6 × 250 mm)—were evaluated under different chromatographic conditions. UV detection was carried out at 254, 272, and 330 nm, with an injection volume of 10 µL. The column temperature is set at 25 °C. Details of the mobile phases and flow rates are provided in Table 5.

Chromatographic performance was assessed by calculating resolution (Rs), symmetry factor (As), number of theoretical plates (N), and retention time (t_R_) for target compounds. Resolution was calculated using the following formula:

Rs = 2(t_R2_ − t_R1_)/(W_1_ + W_2_)

where t_R2_ and t_R1_ represent the retention times of two adjacent peaks, and W_1_ and W_2_ are the respective peak widths at the baseline.

The As was determined using the formula:

As = W_0.05_/2d

where W_0.05_ is the width of the peak at 5% of its height, and d is the distance from the peak start (at 5% height) to the peak maximum. The symmetry factor (As) reflects peak shape: 1.0 = perfect symmetry, <1.0 = fronting, >1.0 = tailing; acceptable values range from 0.8 to 1.8 [28].

The N was calculated using the following formula:

N = 16 × (tR/W)^2^

where N = Number of theoretical plates, tR = Retention time, W = Peak width at baseline

### 4.3. The Forced Degradation Studies

Forced degradation studies were performed to confirm the specificity and to establish its stability-indicating capability. FUR was subjected to acid hydrolysis (1 N HCl, reflux at 60 °C for 3 h), base hydrolysis (1 N NaOH, reflux at 60 °C for 3 h), oxidative degradation (3% H_2_O_2_, reflux at 60 °C for 3 h), thermal degradation (reflux at 60 °C for 3 h), and photolytic stress (direct UV exposure for 3 h). The stressed samples were analyzed using an i-Series LC-2050C 3D chromatograph (Shimadzu Corporation, Nakagyo-ku, Kyoto, Japan).

### 4.4. Method Validation

The analytical method was validated for linearity, accuracy, precision, LOD, and LOQ following the International Council for Harmonisation (ICH) guidelines [18].

#### 4.4.1. Linearity, LOD, and LOQ

Calibration curves were constructed using 7 points of analyte concentrations in standard mixtures of FUR, FUR-B, MP, and PP covering a concentration range of FUR from 2.5 µg/mL to 100 µg/mL, FUR-B from 0.25 µg/mL to 2 µg/mL, MP from 0.31 µg/mL to 30 µg/mL, and PP from 0.25 µg/mL to 7.5 µg/mL. Each concentration was tested three times. Linear regression was used to process the calibration data. Calibration curves were produced by plotting the peak areas vs. the standard concentrations. LOD was determined using the formula of LOD = 3.3σ/S, where σ is the standard deviation of the y-intercepts of the regression lines, and S is the slope of the calibration curve (within the validated linear range for the real sample application). LOQ was calculated using the formula of LOQ = 10σ/S.

#### 4.4.2. Methods of Accuracy and Precision

The accuracy was determined as a percentage recovery by the spiking study, together with the relative standard deviation (%RSD). Three concentrations of standard mixtures of FUR, FUR-B, MP, and PP were added to the sample solution. The spiked sample was prepared in triplicate. The percentage recovery was calculated as Recovery (%) = (Amount detected/Amount added) × 100. Repeatability was assessed by analyzing the samples three times within a single day (*n* = 9), while intermediate precision was determined by performing the analysis over three consecutive days using the same method (*n* = 27). The precision is reported as %RSD (Relative Standard Deviation). %RSD is a measure of precision and is calculated using the formula %RSD (Standard Deviation/Mean) × 100.

#### 4.4.3. Method Robustness and Ruggedness Assessment

The robustness of the developed HPLC method was evaluated by varying critical analytical parameters, including column temperature (23, 25 and 27 °C), flow rate (0.9, 1.0 and 1.1 mL/min), and detection wavelength (270, 270 and 274 nm), to assess the impact on method performance. Ruggedness assessment was performed using different analysts and on different days. Method robustness and Ruggedness were assessed by using RSD of peak area (acceptance criteria ≤ 2%), theoretical plates (>2000), and peak symmetry for all target compounds (0.8–1.8) for FUR, FUR-B, MP, and PP.

### 4.5. Application of a Validated HPLC Method for Demonstration of Method Suitability of Extemporaneous Furosemide Oral Solution

#### 4.5.1. Preparation of Standard and Sample Solutions

Stock solutions of FUR and FUR-B were prepared at concentrations of 500 µg/mL and 62.5 µg/mL, respectively, using a 1:1 (*v*/*v*) mixture of acetonitrile and deionized (DI) water. Stock solutions of MP and PP were prepared at concentrations of 125 µg/mL in DI water. Mixed standard solutions were obtained by combining appropriate volumes of each stock solution and diluting with the mobile phase to the desired concentration ranges of FUR at 2.5–100 µg/mL, FUR-B at 0.25–2.0 µg/mL, MP at 0.3–30 µg/mL, and PP at 0.25–7.5 µg/mL. The calibration range for FUR-B was established to encompass the USP specification limit of ≤ 1.5% relative to labeled furosemide content.

Two formulations were evaluated: furosemide in phosphate buffer (FB) and furosemide in hydroxide solution (FOH) (Table S2). Each formulation was prepared as a 5 mg/mL furosemide oral solution with a batch size of 30 mL. The solutions were dispensed into glass bottles and protected from light throughout the study. Samples were stored under three different conditions: refrigerated (2–8 °C), room temperature (30 ± 2 °C, 75% RH), and accelerated (40 ± 2 °C, 75% RH), during Short-Term Storage (90 days). Samples were withdrawn at predetermined intervals and subjected to chemical and microbial assessment to demonstrate the performance and suitability of the validated HPLC method in real extemporaneous preparations.

#### 4.5.2. Chemical Assessment During Short-Term Storage

Chemical assessment was evaluated at predetermined time points (days 0, 7, 14, 21, 30, 45, 60, 75, and 90) to demonstrate the applicability and performance of the validated HPLC method on real extemporaneous furosemide oral solutions. Each time point, 1 mL of sample (5 mg/mL) was diluted appropriately with the mobile phase and adjusted the volume to 10 mL in a volumetric flask. The solution was then filtered through a 0.22 µm nylon membrane filter prior to HPLC analysis for the determination of FUR, FUR-B, MP, and PP concentrations. Blank solution (0.16 g NaH_2_PO_4_, 3.37 g Na_2_HPO_4_, 14 mL glycerin, 1 g saccharin sodium) was prepared without active ingredients and adjusted to 200 mL with DI water.

#### 4.5.3. Microbial Assessment During Short-Term Storage

Microbial assessment was performed on days 0, 30, 60, and 90 to demonstrate the applicability of the validated HPLC method under typical storage conditions of extemporaneous furosemide oral solutions. Testing was conducted using validated in-house protocols based on pharmacopeial standards for non-sterile products [32], and the dilution method was validated to ensure no effect on microbial viability. Total Aerobic Microbial Count (TAMC) was determined using serial dilution plated on Tryptic Soy Agar (TSA, Merck, Germany) incubated at 30–35 °C for 3–5 days. Yeast and mold counts were determined using Sabouraud Dextrose Agar (SDA, HiMedia, Mumbai, India) plates incubated at 20–25 °C for 14 days. Pathogen detection for *S. aureus*, *P. aeruginosa*, *B. subtilis*, *C. albicans*, *A. brasiliensis*, and *E. coli* used Specific pathogen detection followed compendial methods using appropriate selective media with direct plating and thioglycollate enrichment for confirmatory identification. All procedures were performed in triplicate.

#### 4.5.4. pH Measurement

pH measurements were recorded at each sampling time point using a calibrated pH meter with three replicates per sample.

### 4.6. Statistical Analysis

All experiments were performed in triplicate, and results are presented as mean ± standard deviation (SD). For the application of the validated HPLC method, concentrations of FUR, FUR-B, MP, and PP, as well as microbial counts, were recorded at each time point. Differences among time points were evaluated using one-way analysis of variance (ANOVA) followed by Tukey’s post hoc test, with *p* < 0.05 considered statistically significant. Data analysis and graphical representations were performed using SPSS version 19 (IBM Corporation, Armonk, NY, USA).

## 5. Conclusions

A simple and robust HPLC method was successfully developed and validated for the simultaneous determination of furosemide (FUR), its major degradation product (FUR-B), and two preservatives (MP and PP) in pediatric extemporaneous oral solutions. Chromatographic separation was achieved using a Symmetry^®^ C18 column (5 μm, 4.6 × 250 mm) at 25 °C with a mobile phase consisting of 0.1% acetic acid in DI water:ACN (60:40, *v*/*v*) at a flow rate of 1.0 mL/min. A 10 µL sample volume was injected, and detection was performed at 272 nm. The method showed good specificity, accuracy, precision, and sensitivity and met all validation criteria. Application of the method to stability testing revealed that FUR and preservatives degraded over time under elevated temperatures, with FUR-B increasing accordingly. This method provides a practical tool for quality control and stability monitoring of FUR oral formulations to ensure pediatric safety and efficacy. The developed method provides practical advantages for routine pharmaceutical analysis: it utilizes widely available C18 columns and standard HPLC equipment, employs an isocratic mobile phase that minimizes solvent costs and method complexity, and achieves rapid analysis (<15 min) suitable for high-throughput quality control. These features make the method readily implementable in quality control laboratories without requiring specialized instrumentation or extensive method development.

## Figures and Tables

**Table 1:** Chromatographic Performance Parameters.

Column	Mobile Phase	Flow Rate (mL/min)	Resolution			Symmetry Factor				Number of Theoretical Plates			
			FUR-B→MP	MP→FUR	FUR→PP	FUR-B	MP	FUR	PP	FUR-B	MP	FUR	PP
Kinetex C18	0.1% Acetic acid in DI:ACN (70:30)	0.5	31.68	30.12	45.44	0.73	0.99	1.20	1.20	26,572	38,926	34,123	45,404
	0.1% Acetic acid in DI:ACN (60:40)	0.5	1.85	3.87	4.14	0.8	1.43	2.00	1.58	5733	8222	1782	2835
Symmetry^®^ C18	0.1% Acetic acid in DI:ACN (70:30)	1	20.77	21.43	18.83	1.17	2.94	2.17	1.15	11,855	21,424	17,485	22,347
	0.1% Acetic acid in DI:ACN (60:40)	1	17.52	10.24	16.81	1.18	1.45	1.12	0.99	20,745	23,447	17,224	18,200

**Table 2:** Forced degradation results and peak purity assessment.

Degradation Condition	Degradation (% Assay Loss)	Major Degradant (FUR-B) (% Area)	Minor Degradants (% Area)	%Mass Balance	Peak Purity Index
Acid stress	8.93	7.03	1.81	99.91	0.930598
Base stress	0.82	0.10	0.62	99.90	0.958154
Oxidation stress	15.58	8.86	6.64	99.91	0.957837
Thermal stress	0.90	0.11	0.69	99.90	0.958991
Photolysis	0.37	0.10	0.17	99.90	0.960242

**Table 3:** The linearity range, LOD, and LOQ of FUR, FUR-B, MP, and PP.

Analytical Characteristics	FUR	FUR-B	MP	PP
Linear Range (µg/mL)	2.50–100.0	0.25–2.00	0.31–30.00	0.25–7.50
R^2^	0.9992	0.9997	0.9997	0.9995
LOD (µg/mL)	1.14	0.12	0.15	0.08
LOQ (µg/mL)	3.45	0.36	0.44	0.24

**Table 4:** Intra-day and inter-day accuracy and precision of FUR, FUR-B, MP, and PP.

Compound	Concentration (µg/mL)	Intra-Day (*n* = 9)			Inter-Day (*n* = 27)		
		Measured Concentration (µg/mL)	Accuracy	Repeatability	Measured Concentration (µg/mL)	Accuracy	Intermediate Precision
			Recovery (%)	RSD (%)		Recovery (%)	RSD (%)
FUR	25.00	24.87 ± 0.24	99.5 ± 1.0	1.0	24.89 ± 0.24	99.6 ± 1.0	1.0
	50.00	49.93 ± 0.28	99.9 ± 0.6	0.6	49.95 ± 0.25	99.9 ± 0.5	0.5
	75.00	74.58 ± 0.44	99.4 ± 0.6	0.6	74.36 ± 0.49	99.2 ± 0.7	0.7
FUR-B	0.75	0.75 ± 0.01	100.2 ± 1.5	1.5	0.75 ± 0.01	100.6 ± 1.8	1.8
	1.00	1.01 ± 0.01	101.0 ± 0.8	0.8	1.01 ± 0.02	100.9 ± 1.5	1.5
	1.50	1.50 ± 0.02	100.0 ± 1.1	1.1	1.50 ± 0.02	99.9 ± 1.0	1.1
MP	5.00	4.95 ± 0.01	99.1 ± 0.1	0.1	4.99 ± 0.06	99.7 ± 1.2	1.3
	10.00	10.00 ± 0.06	100.0 ± 0.6	0.6	10.02 ± 0.06	100.2 ± 0.6	0.6
	15.00	14.92 ± 0.04	99.5 ± 0.3	0.3	14.97 ± 0.12	99.8 ± 0.8	0.8
PP	1.00	1.00 ± 0.01	100.4 ± 0.9	0.9	1.01 ± 0.02	100.5 ± 1.8	1.7
	2.00	1.98 ± 0.03	99.2 ± 1.4	1.4	1.96 ± 0.03	98.2 ± 1.6	1.6
	3.00	2.96 ± 0.04	98.8 ± 1.2	1.2	2.99 ± 0.06	99.8 ± 2.0	2.0

**Table 5:** Chromatographic conditions for HPLC analysis.

Column	Mobile Phase Composition	Flow Rate	Detection Wavelength
Kinetex C18 (2.6 μm, 4.6 × 150 mm)	0.1% acetic acid in DI water: acetonitrile (70:30)	0.5 mL/min	254, 272, and 330 nm
	0.1% acetic acid in DI water: acetonitrile (60:40)	0.5 mL/min	254, 272, and 330 nm
Symmetry^®^ C18 (5 μm, 4.6 × 250 mm)	0.1% acetic acid in DI water: acetonitrile (70:30)	1.0 mL/min	254, 272, and 330 nm
	0.1% acetic acid in DI water: acetonitrile (60:40)	1.0 mL/min	254, 272, and 330 nm

## Data Availability

The data presented in this study is available on request from the corresponding author.

## Supplementary Materials

The following supporting information can be downloaded at: https://www.mdpi.com/article/10.3390/molecules30194031/s1, Figure S1: HPLC chromatograms of FUR subjected to acid, base, oxidative, thermal, and photolytic stress conditions; Table S1: Retention time of the optimized condition; Table S2: Composition and quantity of ingredients in pediatric extemporaneous furosemide oral solution; Table S3: Robust evaluation of the developed HPLC method under varied chromatographic conditions; Table S4: The ruggedness testing results of the analytical method under varying chromatographic conditions; Table S5: The statistical parameters of the sigmoidal curve fit, along with the 95% confidence intervals of the degradation trends; Table S6: pH stability of furosemide oral formulations during 90-day storage study.
