# Supplementary material for: Simultaneous Development and Validation of an HPLC Method for the Determination of Furosemide and Its Degraded Compound in Pediatric Extemporaneous Furosemide Oral Solution
Source: Molecules. 2025 Oct 9;30(19):4031. doi: 10.3390/molecules30194031 (PMC12526389; doi:10.3390/molecules30194031)
Supplement: Supplementary file 1 [file molecules-30-04031-s001.zip › molecules-3833147-supplementary.pdf]

molecules

MDPI

Article

Simultaneous Development and Validation of an HPLC Method for the Determination of Furosemide and Its Degraded Compound in Pediatric Extemporaneous Furosemide Oral Solution

Katsanee Srejomthong [1,2], Thanawat Pattananandecha [2,3], Sutasinee Apichai [2,3], Suporn Charumanee [1,2], Busaban Sirithunyalug [1,2], Fumihiko Ogata [4], Naohito Kawasaki [4,5] and Chalermpong Saenjum [1,3,*]

1 Department of Pharmaceutical Sciences, Faculty of Pharmacy, Chiang Mai University, Chiang Mai 50200, Thailand; katsanee.s@cmu.ac.th (K.S.); chsuporn@gmail.com (S.C.); busaban.s@cmu.ac.th (B.S.)

2 Research Center for Innovation in Analytical Science and Technology for Biodiversity-Based Economic and Society (I-ANALY-S-T_B.BES-CMU), Multidisciplinary Research Institute (MDRI), Chiang Mai University, Chiang Mai 50200, Thailand; thanawat.pdecha@gmail.com (T.P.); sutasinee.apichai@gmail.com (S.A.)

3 Office of Research Administration, Chiang Mai University, Chiang Mai 50200, Thailand

4 Faculty of Pharmacy, Kindai University, 3-4-1 Kowakae, Higashi-Osaka 577-8502, Osaka, Japan; ogata@phar.kindai.ac.jp (F.O.); kawasaki@phar.kindai.ac.jp (N.K.)

5 Antiaging Center, Kindai University, 3-4-1 Kowakae, Higashi-Osaka 577-8502, Osaka, Japan

* Correspondence: chalermpong.s@cmu.ac.th; Tel.: +66-89-950-4227

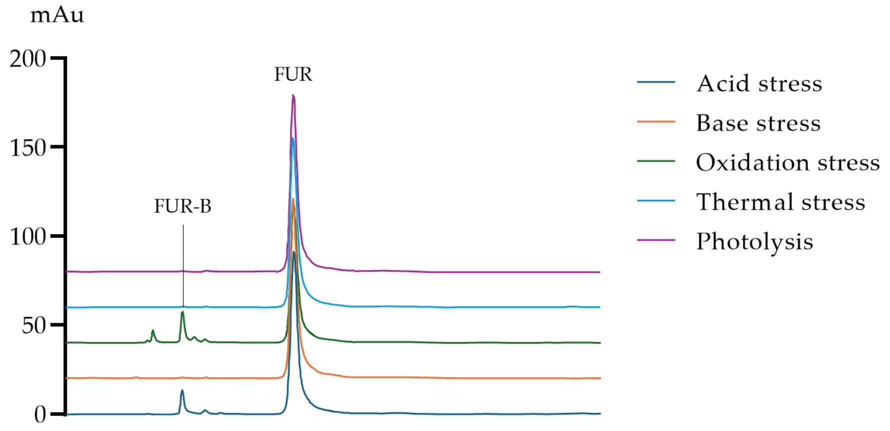

Figure S1. HPLC chromatograms of FUR subjected to acid, base, oxidative, thermal, and photolytic stress conditions.

 https://doi.org/10.3390/xxxxx

Table S1. Retention time of the optimized condition.

Column	Mobile phase	Flow rate	Retention time (min)			
			FUR-B	MP	FUR	PP
Kinetex C18	0.1% Acetic acid in DI water:ACN (70:30)	0.5	4.04	8.27	16.02	28.19
	0.1% Acetic acid in DI water:ACN (60:40)	0.5	3.41	3.73	4.95	7.23
Symmetry® C18	0.1% Acetic acid in DI water:ACN (70:30)	1	3.78	7.3	13.97	24.04
	0.1% Acetic acid in DI water:ACN (60:40)	1	3.28	5.29	7.10	11.88

FUR-B: Furosemide-related compound B (2-amino-4-chloro-5-sulfamoylbenzoic acid); MP: Methylparaben; FUR: Furosemide; PP: Propylparaben; ACN: Acetonitrile; DI: Deionized water. All experiments were performed at 25 °C with detection at 272 nm.

Table S2. Composition and quantity of ingredients in pediatric extemporaneous furosemide oral solution.

Ingredient	Furosemide in phosphate buffer (FB)	Furosemide in hydroxide solution (FOH)
Furosemide (g)	1	1
Sodium dihydrogen phosphate dihydrate (g)	0.16	-
Disodium hydrogen phosphate dihydrate (g)	3.37	-
0.2 M Sodium hydroxide (mL)	-	16.5
Glycerin (mL)	14.0	14.0
Saccharin sodium (g)	1	1
Concentrated parabens solution (mL)	2.0	2.0
1 M Sodium hydroxide (q.s. to pH 8)	8	8
Purified water q.s. to (mL)	200	200

Table S3. Robust evaluation of the developed HPLC method under varied chromatographic conditions.

Chromatographic condition	Mixed Standard											
	FUR-B			MP			FUR			PP		
	RSD (%) of Peak Area	Theoretical plates	Peak symmetry	RSD (%) of Peak Area	Theoretical plates	Peak symmetry	RSD (%) of Peak Area	Theoretical plates	Peak symmetry	RSD (%) of Peak Area	Theoretical plates	Peak symmetry
Proposed HPLC condition	1.31	12343	0.98	0.34	13107	1.18	0.32	9359	1.21	0.91	22472	1.03
**Column temperature**												
23 °C	0.96	13567	1.01	0.39	13376	1.17	0.17	8776	1.31	1.34	22331	1.04
27 °C	0.39	12917	1.02	0.47	13940	1.03	0.77	9839	1.25	0.75	23187	1.02
**Flow rate**												
0.9 mL/min	1.73	12308	1.23	0.71	13350	1.28	0.21	9609	1.36	1.97	22982	1.15
1.1 mL/min	0.73	12735	1.05	0.50	13257	1.36	0.80	9054	1.27	1.63	22753	1.01
**Detection wavelength (nm)**												
270 at Flow rate 1.0 mL/min	1.09	12489	1.03	0.27	12764	1.34	0.39	9237	1.42	1.91	22884	1.09
274 at Flow rate 1.0 mL/min	1.01	13047	1.02	0.28	13008	1.27	0.93	9324	1.32	0.81	23285	1.05

Mixed standard: FUR (20 μg/mL), FUR-B (0.75 μg/mL), MP (7.5 μg/mL), and PP (0.5 μg/mL).

Table S3. Robust evaluation of the developed HPLC method under varied chromatographic conditions (continued).

Chromatographic condition	Mixed Standard											
	FUR-B			MP			FUR			PP		
	RSD (%) of Peak Area	Theoretical plates	Peak symmetry	RSD (%) of Peak Area	Theoretical plates	Peak symmetry	RSD (%) of Peak Area	Theoretical plates	Peak symmetry	RSD (%) of Peak Area	Theoretical plates	Peak symmetry
**Volume of Injection (μL)**												
8 μL	1.92	12404	1.33	1.70	13592	1.28	1.70	9592	1.38	1.53	22927	1.32
12 μL	0.72	13093	1.21	0.92	13854	1.34	1.23	9633	1.39	1.78	22993	1.21
**Mobile phase**												
0.1% acetic acid in water and acetonitrile (62:38, v/v)	0.47	12696	1.33	0.24	13370	1.08	1.45	10560	1.22	1.53	22073	1.09
0.1% acetic acid in water and acetonitrile (58:42, v/v)	0.24	13354	1.04	0.06	12762	1.33	0.57	10431	1.34	1.42	21521	1.25

Table S4. The ruggedness testing results of the analytical method under varying chromatographic conditions.

Chromatographic condition	Mixed Standard											
	FUR-B			MP			FUR			PP		
Column temperature: 25°C<br>Flow rate: 1.0 mL/min at 272 nm<br>Injection volume: 10 µL<br>Mobile phase:<br>0.1% acetic acid in water and acetonitrile (60:40, v/v)	RSD (%) of Peak Area	Theoretical plates	Peak symmetry	RSD (%) of Peak Area	Theoretical plates	Peak symmetry	RSD (%) of Peak Area	Theoretical plates	Peak symmetry	RSD (%) of Peak Area	Theoretical plates	Peak symmetry
**Analyst**												
HPLC Model I	0.47	12598	1.04	0.18	12895	1.32	0.15	9775	1.35	0.18	22257	1.31
HPLC Model II	0.24	13515	1.01	0.05	13888	1.28	0.04	10134	1.28	0.01	21658	1.12
**Inter Day**												
Day 1	0.24	13515	1.01	0.05	13888	1.28	0.04	10134	1.28	0.01	21658	1.12
Day 2	0.92	13284	0.99	0.08	13491	1.29	0.06	9543	1.33	0.99	22818	1.30

Mixed standard: FUR (50 µg/mL), FUR-B (1.0 µg/mL), MP (10 µg/mL), and PP (2.0 µg/mL).

Table S5. The statistical parameters of the sigmoidal curve fit, along with the 95% confidence intervals of the degradation trends.

Formulation		Storage	Statistical parameters	
			HillSlope	R squared
FB	FUR	2-8 °C	-5.057±0.645	0.9739±0.0178
		30 °C/75% RH	-1.445±0.099	0.8991±0.0168
		40 °C/75% RH	-2.883±0.188	0.9567±0.0052
	MP	2-8 °C	-0.978±0.008	0.9185±0.0240
		30 °C/75% RH	-1.869±0.176	0.9551±0.0075
		40 °C/75% RH	-0.810±0.170	0.9896±0.0056
	PP	2-8 °C	-1.699±0.012	0.9375±0.0340
		30 °C/75% RH	-0.967±0.143	0.9323±0.0313
		40 °C/75% RH	-1.724±0.129	0.9596±0.0102
FOH	FUR	2-8 °C	-8.228±0.664	0.9579±0.0108
		30 °C/75% RH	-3.738±0.169	0.9387±0.0105
		40 °C/75% RH	-1.818±0.260	0.8529±0.0324
	MP	2-8 °C	-1.253±0.165	0.9304±0.0309
		30 °C/75% RH	-1.585±0.018	0.9444±0.0061
		40 °C/75% RH	-1.000±0.169	0.9455±0.0189
	PP	2-8 °C	-0.506±0.244	0.7349±0.0329
		30 °C/75% RH	-2.206±0.332	0.9240±0.0163
		40 °C/75% RH	-1.318±0.088	0.9525±0.0031

Table S6. pH stability of furosemide oral formulations during 90-day storage study**.**

Day	FB formulation			FOH formulation		
	**2-8 °C**	**30 °C/75% RH**	**40 °C/75% RH**	**2-8 °C**	**30 °C/75% RH**	**40 °C/75% RH**
0	8.03 ± 0.01	8.03 ± 0.01	8.03 ± 0.01	8.04 ± 0.03	8.04 ± 0.03	8.04 ± 0.03
7	8.03 ± 0.01	8.02 ± 0.01	8.02 ± 0.01	8.02 ± 0.01	8.05 ± 0.01	8.01 ± 0.01
14	8.02 ± 0.01	7.96 ± 0.00	7.97 ± 0.01	8.00 ± 0.01	7.95 ± 0.02	7.93 ± 0.02
21	7.96 ± 0.01	7.94 ± 0.01	7.92 ± 0.01	7.96 ± 0.01	7.88 ± 0.01	7.78 ± 0.01
30	7.98 ± 0.01	7.94 ± 0.01	7.91 ± 0.01	7.97 ± 0.01	7.89 ± 0.01	7.77 ± 0.01
45	7.96 ± 0.01	7.92 ± 0.01	7.88 ± 0.01	7.94 ± 0.01	7.83 ± 0.01	7.68 ± 0.00
60	7.94 ± 0.01	7.90 ± 0.01	7.85 ± 0.00	7.93 ± 0.01	7.74 ± 0.01	7.56 ± 0.02
75	7.95 ± 0.01	7.91 ± 0.01	7.84 ± 0.01	7.94 ± 0.01	7.72 ± 0.01	7.54 ± 0.01
90	7.94 ± 0.00	7.89 ± 0.01	7.82 ± 0.01	7.83 ± 0.01	7.70 ± 0.01	7.46 ± 0.00
